# Peer Review of “The Exchange of Informational Support in Online Health Communities at the Onset of the COVID-19 Pandemic: Content Analysis”

**DOI:** 10.2196/31423

**Published:** 2021-07-22

**Authors:** Reem El Sherif

**Affiliations:** 1 Department of Family Medicine McGill University Montreal, QC Canada

**Keywords:** COVID-19, health information, informational support, online health, online health communities, online platform, pandemic, social support

This is a peer-review report submitted for the paper “The Exchange of Informational Support in Online Health Communities at the Onset of the COVID-19 Pandemic: Content Analysis”.

## Round 1 Review

### General Comments

This paper [1] describes an interesting and very important study on the contents of an online health community (OHC) on COVID-19. The authors conducted a content analysis of the community posts in an online health information platform and provide recommendations for public health responses during this, and future, pandemics. I believe this is very important work.

I provide some feedback that would potentially strengthen the paper and improve its readability for the journal audience.

### Specific Comments

#### Major Comments

1. There are some issues with the references: the order needs to be revised, eg, the first reference is number 8.

2. In the fourth paragraph of the introduction (starting with “Although social support…”), there are no references to which definitions of social support or information needs or information seeking are used by the authors. In fact, there appears to be some overlap between these three concepts in this paragraph, while they are actually three distinct concepts in the literature. I would suggest the authors familiarize themselves with some of the seminal work on information-seeking behavior by Wilson and Bates and on social support by Tardy and Barrera:

Barrera, M. Distinctions between social support concepts, measures, and models. Am J Community Psychol 1986; 14(4):413-445.

Bates, M. Toward an integrated model of information seeking and searching. 2002 Presented at: Fourth International Conference on Information Needs, Seeking and Use in Different Contexts; September 11, 2002; Lisbon, Portugal p. 1-15.

Tardy, CH. Social support measurement. Am J Community Psychol 1985; 13(2):187-202.

Wilson, T. Models in information behaviour research. J Doc 1999; 55(3):249-270. [doi:10.1108/EUM0000000007145]

3. The section titled *Prior Work* was difficult to read; it lacks organization and coherence. I was unsure what points the authors were making since it seemed to be just a summary of the existing literature without any synthesis of the findings. Perhaps this section can be divided into two subsections: “Social support in OHCs” and “Information needs during the pandemic,” or something similar. The authors can identify the clear knowledge gaps at the end that their study is addressing.

4. In the *Methods* section, can the authors provide some detail on who did the coding and how the codebooks in Tables 1 and 2 were developed? It is only in the *Limitations* section that we discover it was one coder; were other researchers perhaps involved in the development of the codebook, was it tested and revised, was the coding checked, etc?

5. In the *Results* section, the authors state “Those who were in a position to offer information had a significantly higher percentage of responding more than once (P < 0.001).” Can they provide more explanation on how they defined “being in a position to offer information” and how the information was derived from the posts or user profiles?

6. Were there any incidences of emotional support in the posts? Their presence (or lack thereof) would be an interesting point to add if possible.

7. In the *Discussion* section, it may be interesting to contrast these findings with those reported in other studies in different contexts.

#### Minor Comments

8. The whole paper might benefit from professional editorial revision. In the first paragraph of the Introduction, for example, I would suggest revising “trauma in the” to “trauma among healthcare workers” and revising “becomes” to “become increasingly important”.

9. On page 5, “namely sliding-ONMF and rolling-ONMF” is used with no explanation.

10. In the *Methods* section, a short summary of MedHelp would perhaps be helpful for the journal’s international readers.

11. Do the authors perhaps mean “Types of information seeking topics” for Table 1?

12. Perhaps the authors can reference the method they used for analysis (qualitative content analysis)?

## Round 2 Review

### General Comments

The authors have addressed all the previous comments made, and the paper is much more coherent and relevant. I especially appreciate the additional section on prior work and the detail added to the methods section for clarity.

The manuscript may still require some professional editing; there are some minor grammatical errors that could be addressed.

## Abbreviations

OHC: online health community
